# Patients who do not fulfill criteria for hypertrophic cardiomyopathy but have unexplained giant T-wave inversion: a cardiovascular magnetic resonance mid-term follow-up study

**DOI:** 10.1186/s12968-020-00700-5

**Published:** 2021-06-03

**Authors:** Shuang Li, Jian He, Jing Xu, Baiyan Zhuang, Bailing Wu, Bingqi Wei, Jinghan Huang, Gang Yin, Xiuyu Chen, Zhenhui Zhu, Hao Wang, Shihua Zhao, Minjie Lu

**Affiliations:** 1 Department of Magnetic Resonance Imaging, State Key Laboratory of Cardiovascular Disease, Fuwai Hospital, National Center for Cardiovascular Diseases, Chinese Academy of Medical Sciences and Peking Union Medical College, 167 Beilishi Road, Xicheng District, Beijing, 100037 China; 2 Department of Radiology, The Second Hospital of Hebei Medical University, Shijiazhuang, Hebei China; 3 Department of Cardiology, State Key Laboratory of Cardiovascular Disease, Fuwai Hospital, National Center for Cardiovascular Diseases, Chinese Academy of Medical Sciences and Peking Union Medical College, Beijing, China; 4 The Heart-Lung Testing Center, State Key Laboratory of Cardiovascular Disease, Fuwai Hospital, National Center for Cardiovascular Diseases, Chinese Academy of Medical Sciences and Peking Union Medical College, Beijing, China; 5 Department of Echocardiography, State Key Laboratory of Cardiovascular Disease, Fuwai Hospital, National Center for Cardiovascular Diseases, Chinese Academy of Medical Sciences and Peking Union Medical College, Beijing, China; 6 Key Laboratory of Cardiovascular Imaging (Cultivation), Chinese Academy of Medical Sciences, Beijing, 100037 China

**Keywords:** Follow-up study, CMR, Hypertrophic cardiomyopathy, Giant T-wave inversion, Segmental wall thickness

## Abstract

**Background:**

Patients who have unexplained giant T-wave inversions but do not meet criteria for hypertrophic cardiomyopathy (HCM) (left ventricular (LV) wall thickness < 1.5 cm) demonstrate LV apical morphological features that differ from healthy subjects. Currently, it remains unknown how the abnormal LV apical morphology in this patient population changes over time. The purpose of this study was to investigate LV morphological and functional changes in these patients using a mid-term cardiovascular magnetic resonance (CMR) exam.

**Methods:**

Seventy-one patients with unexplained giant T-wave inversion who did not fulfill HCM criteria were studied. The mean interval time of the follow-up CMR was 24.4 ± 8.3 months. The LV wall thickness was measured in each LV segment according to the American Heart Association 17-segmented model. The apical angle (ApA) was also measured. A receiver operating curve (ROC) was used to identify the predictive values of the CMR variables.

**Results:**

Of 71 patients, 16 (22.5%) progressed to typical apical HCM, while 55 (77.5%) did not progress to HCM criteria. The mean apical wall thickness was significantly different between the two groups at both baseline and follow-up, with the apical HCM group having greater wall thickness at both time points (all *p* < 0.001). There was a significant difference between the two groups in the change of ApA (− 1.5 ± 2.7°/yr vs. − 0.7 ± 2.0°/yr, *p* < 0.001) over time. The combination of mean apical wall thickness and ApA proved to be the best predictor for fulfilling criteria for apical HCM with a threshold value of 8.1 mm and 90° (sensitivity 93.8%, specificity 85.5%).

**Conclusions:**

CMR metrics identify predictors for progression to HCM in patients with unexplained giant T-wave inversion.

**Supplementary information:**

The online version contains supplementary material available at 10.1186/s12968-020-00700-5.

## Introduction

Apical hypertrophic cardiomyopathy (HCM), a variant of HCM [1, 2], is common in East Asia and accounts for up to 41% of all HCM cases [3, 4]. An “ace of spades” configuration of the left ventricular (LV) cavity on imaging studies such as cardiovascular magnetic resonance (CMR) [5] and giant negative T wave inversions on the electrocardiogram (ECG) [6] are both typical characteristics of apical HCM. Although apical HCM has a relatively benign prognosis in terms of cardiovascular mortality, up to 25% of individuals can go on to develop significant late cardiovascular events which include heart failure, chest discomfort, apical fibrosis, apical aneurysm formation (with or without intracavitary obstruction), stroke, atrial fibrillation, and ventricular tachycardia [3–5].

As of now, the diagnostic criteria of HCM is defined by LV wall thickness ≥ 15 mm (≥ 13 mm with HCM family history). This diagnostic criteria for HCM was published by American Heart Association (AHA)/American College of Cardiology Foundation (ACCF) in 2011 [7] and subsequently by the European Society of Cardiology (ESC) in 2014 [8]. Several previous studies, including our own prior CMR study, have reported that the normal LV wall thickness thins as it progresses from the base to apex. Thus, LV wall thickness in the apical segments should be less than that of the basal segments [5, 9, 10]. Our initial study found that patients who did not fulfill CMR criteria for HCM but who had unexplained giant T-wave inversion on ECG had abnormal LV apical morphology. Specifically, even though these patients did not have an LV wall thickness of ≥ 15 mm, they had abnormal morphology at the LV apex such as lack of the normal thinning of the LV wall in the apex relative to the base [9]. However, it was unknown what proportion of the patients in this population would go on to fulfill  criteria for apical HCM and what are the predictors for progression. In this context, we performed a follow-up CMR study to evaluate the morphological changes and outcomes in this cohort to assess the main imaging determinants that predict the evolution to apical HCM.

## Methods

### Study population

Consecutive subjects with unexplained ECG giant T-wave inversion from January 2006 to December 2017 were retrospectively identified. Patients who met all of the following inclusion criteria were enrolled in this study: (1) standard 12-lead ECG with deep T-wave inversion, most prominent in the anterolateral leads (V3–V5 leads) with the negative T wave voltage ≥ 5 mm, (2) Non-obstructive coronary artery disease (< 50% narrowing of a coronary artery secondary to plaque) on coronary computed tomography or invasive coronary angiography, (3) end-diastolic apical wall thickness < 15 mm (< 13 mm if family history of HCM), and (4) at least two CMR examinations were performed, and the minimum interval between two CMR examinations was at least 6 months. Subjects were routinely excluded if they had one of the followings conditions: (1) non-sinus rhythm, (2) T-wave inversion in ≤ 2 contiguous leads, concomitant bundle branch block or QRS > 80 ms or QTC > 440 ms, (3) hypertension (systolic blood pressure ≥ 140 mmHg and/or diastolic pressure ≥ 90 mmHg on two or more consecutive visits without anti-hypertensive medications), (4) severe valvular lesions, pericardial disease, cardiac tumor, immunological or metabolic disease involving heart, (5) history of cardiac surgery. The study protocol was approved by the institutional review board of our hospital and written informed consent was waived. The study was conducted in accordance with the Declaration of Helsinki.

### CMR scanning protocols

CMR exams were performed on three different CMR scanners: a 1.5 T scanner (Avanto, Siemens Healthineers, Erlangen, Germany; 3 T MR750 (General Electric Healthcare, Waukesha, Wisconsin, USA; or 3 T Ingenia (Philips Healthcare, Best, the Netherlands). A three-lead vector cardiogram was used for ECG gating. A complete short-axis stack, 4-chamber and 2-chamber balanced steady state free precession cine images of the LV were acquired using retrospective ECG-gating. Sequence parameters included: slice thickness: 6–8 mm, slice gap 30–50% wall thickness; matrix: (156–256) × (192–256); flip angle: 80°; parallel acquisition technique factor: 2; and bandwidth: 930 Hz/PX. A phase-sensitive inversion-recovery gradient-echo pulse sequence with coil intensity correction (FOV 320–360 × 250 mm^2^, matrix of 134 × 256, time to repetition/time to echo of RR interval/3.38 ms; FA 35◦, slice thickness of 6 mm) was used for the late gadolinium enhancement(LGE) imaging, which was acquired approximately 10–15 min after a 0.2 mmol/kg intravenous dose of gadolium (gadopentate dimeglumine, Magnevist, Bayer Healthcare, Berlin, Germany) during breath hold in a series of short-axis planes and four- and two-chamber long-axis planes [11, 12].

### CMR image analysis

CMR images were transferred to a commercial off-line workstation for further analysis using Qmass® (version 7.6, Medis Medical Imaging Systems, Inc. Leiden, the Netherlands). LV wall thickness was manually measured in all 16 segments at the end-diastolic phase. The true apex was excluded (17th segment). These segmental LV wall thickness measurements were performed as detailed in our previous publication (see Additional file 1) [9]. Briefly, the basal wall thickness measurements were made approximately 1.5 cm away from the atrioventricular junctions, mid-cavity wall thickness measurements were made using short-axis images at the level of the papillary muscles, and apical wall thickness measurements were made 2 cm distance from the true apex on 2-chamber and 4-chamber long axis views. The apical angle (ApA) was also measured as described in our previous study [9]. Other global morphological and functional measures derived from CMR were also measured or calculated. The left atrial (LA) and LV dimensions were measured as described previously [13, 14]. In detail, the LA dimension was measured at end-systole on the three-chamber cine in order to obtain a maximum diameter of the LA cavity. LV cavity dimensions were measured on the short axis cines at the papillary muscle tip level at end diastole. LV ejection fraction (LVEF), LV end-diastolic volume indexed to body surface area (BSA) (LVEDVI), LV end-systolic volume indexed to BSA (LVESVI), and cardiac index (CI) were obtained by Argus ® (VB15, Siemens Healthineers). LGE was defined as an image intensity level > 6 SD above the mean of image intensities in a remote basal segment in the same image [15, 16]. The location of enhanced myocardium was analyzed in a 17-segment model (excluding apex) based on short-axis views. The number of positive segments and the LGE volume were calculated and used for further analysis.

The patients were divided into two groups based on the absolute apical wall thickness as well as the increase in percentage of apical wall thickness at follow-up CMR. Group 1: typical apical HCM defined as patients whose apical LV wall thickness increased from baseline and now fulfilling diagnostic criteria for apical HCM. Group 2: pre-apical HCM defined as patients whose apical LV wall thickness increased compared to baseline, but still do not meet the diagnostic criteria for apical HCM.

All CMR images were analyzed by two experienced radiologists, who were blinded to the echocardiographic, ECG and clinical data. Interobserver and intraobserver variability were tested in a sub-group of randomized selected 30 subjects.

### Statistical analysis

Continuous variables with normal distribution were expressed as mean ± standard deviation. Category variables were presented as numbers (proportion) and differences between groups and were analyzed by Fisher’s exact test or chi-square test. The Student paired *t* test was used to analyzed the wall thickness between baseline and follow-up. Rate of change in the CMR parameters was also calculate and analyzed by the Student t test. LV segmental wall thickness differences between groups were also analyzed by the student t test. The Mann–Whitney U test was used to analyze non-normally distributed variables. The correlation between the change of wall thickness and time interval between two CMR examinations was analyzed by simple linear regression analysis and Pearson’s correlation analysis. A receiver operating curve (ROC) was used to identify the predictive values of the CMR variables. Univariable Cox regression models were used to estimate the unadjusted hazard prediction of apical HCM. Hazard ratios were generated and expressed together with their 95% confidence intervals (CIs). All data were analyzed using SPSS (version 22.0, Statistical Package for the Social Sciences, International Business Machines, Inc., Armonk, New York, USA). Intraclass correlation coefficients and Bland–Altman plots were used to assess intra- and interobserver variability by using SPSS and GraphPad (version 6.0, Graph-Pad Software, La Jolla, California, USA), respectively [17]. A two-tailed values of  *p*< 0.05 were regarded as statistically significant.

## Results

### Clinical characteristics

**Fig. 1:**
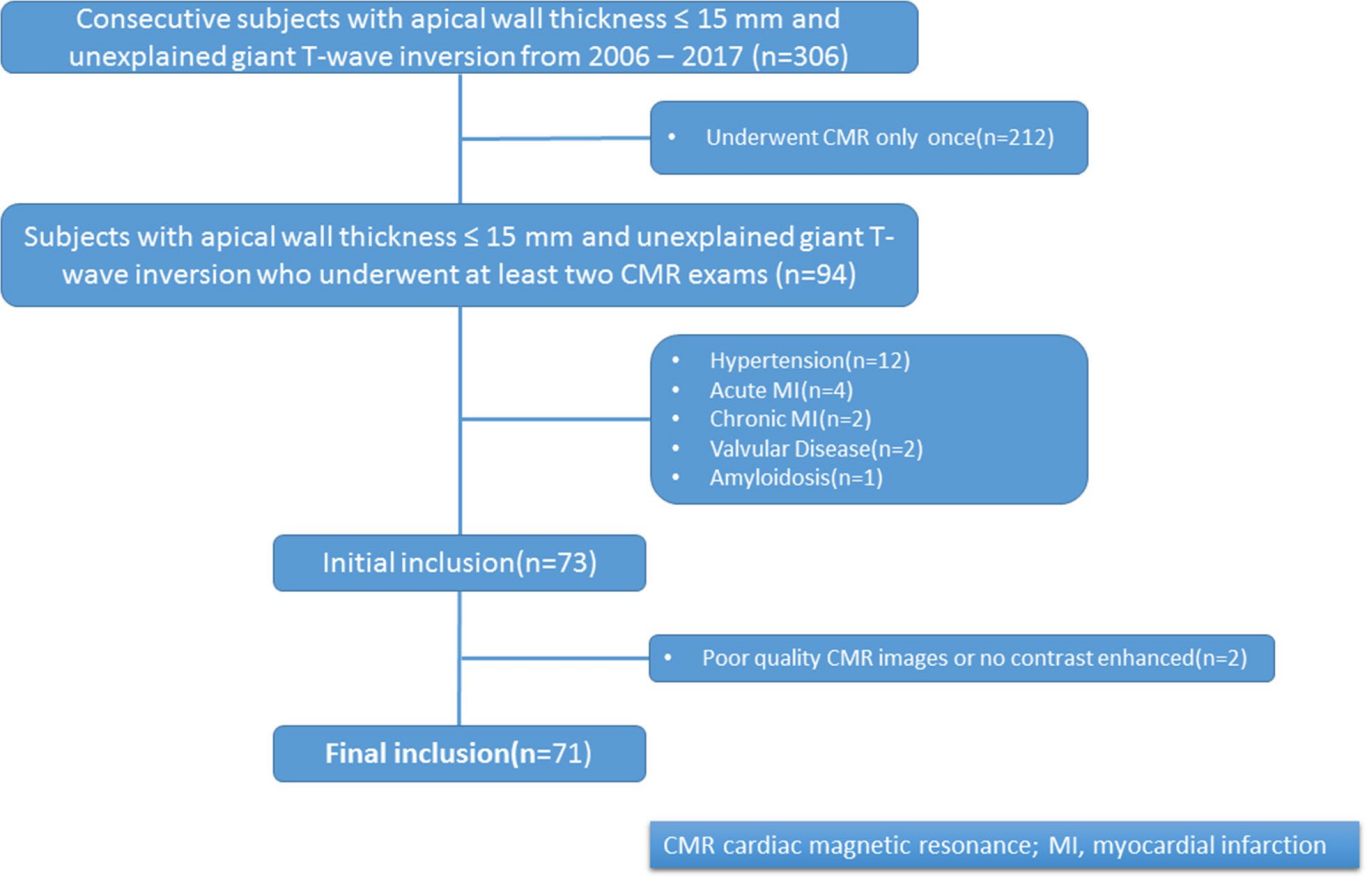
The flow chart depicts patient enrollment secondary to inclusion and exclusion criteria. Patients’ selection process and exclusion criteria. *HCM* hypertrophic cardiomyopathy, *CMR* cardiovascular magnetic resonance

Three hundred and six patients fulfilled the initial study inclusion criteria of which 212 were excluded due to absence of a follow-up CMR examination. Another 21 patients were excluded for hypertension (n = 12), acute myocardial infarction (n = 4), chronic myocardial infarction (n = 2), valvular disease (n = 2) and amyloidosis (n = 1). The remaining 71 patients (61 (85.9%) males; 49.4 ± 12.9 years) were enrolled (Fig. 1). The mean time interval between the baseline and follow-up CMR examinations was 24.4 ± 8.3 months (range = 9 to 48 months). The mean time interval between two CMR examinations was longer in group 1 (33.9 ± 6.4 months, range = 27 to 48 months vs. 24.8 ± 5.7 months, range = 16 to 41 months).

**Fig. 2:**
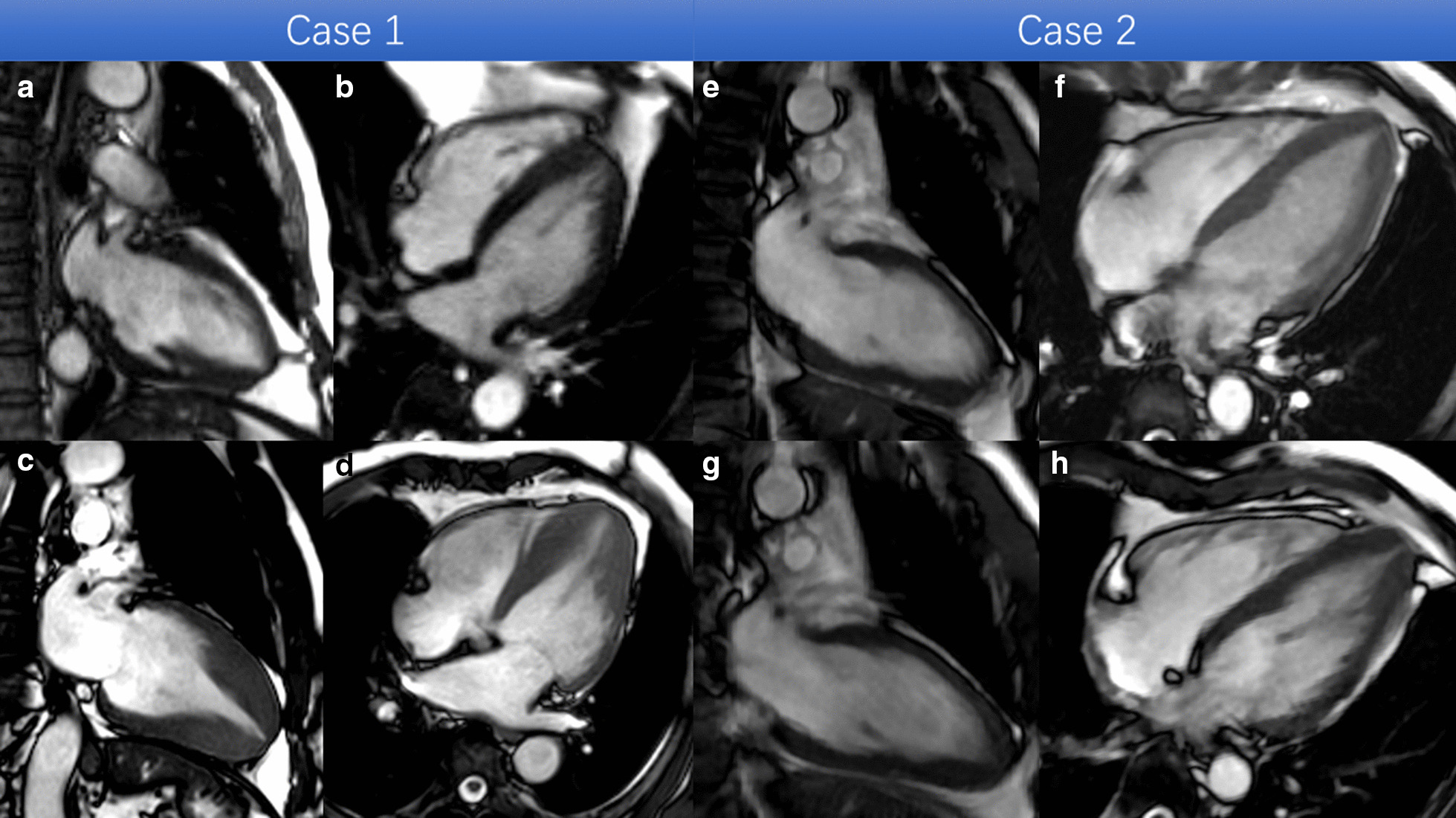
Representative cases of patients with unexplained giant T-wave inversions on electrocardiogram (ECG). Patient 1 (**a**–**d**) a 59-year-old male and is a typical case from Group 1. **a** and **b** show the two chamber and 4 chamber end-diastolic balanced steady state free precession (bSSFP) cine images respectively at baseline. **c** and **d **were the corresponding 2 and 4 chamber end-diastolic bSSFP cine images at follow-up 48 months later when this patient fulfilled criteria for apical HCM. Patient 2 (**e**–**h**) is a 57 years-old male and represents a typical case from Group 2. **e** and **f** show the 2 chamber and 4 chamber end-diastolic bSSFP cine phases respectively at baseline. **f** and **g** show the 2 and 4 chamber cine views, respectively, 32 months later. The wall thickness of apex has become became (average 35% increased) when compared to baseline, however the absolute value does not meet the diagnostic criteria for apical HCM

The cohort was divided into two groups based on the absolute apical wall thickness as well as increased percentage of apical wall thickness at follow-up CMR. Group 1: typical apical HCM, n = 16 (22.5%) met criteria for apical HCM at follow-up (group 1, Fig. 2a–d). Group 2: pre-apical HCM, n = 55 (77.5%) who did not meet LV wall thickness criteria for apical HCM (group 2, Fig. 2e–h). There were more symptoms (*p* < 0.001) and more ECG abnormalities (ST-T abnormality and LV hypertrophy) in group 1 (both  *p* < 0.001). Of the 16 patients in group 1, three patients were diagnosed as apical HCM and 7 were diagnosed as suspicious for apical HCM on echocardiography. However, there was only one group 2 subject diagnosed as suspicious for apical HCM. There were no differences in other baseline characters between two groups. The detailed baseline clinical characteristics of the patients were listed in Table 1.

**Table 1:** Baseline characteristics of this cohort

	Patients (n = 71)	Group 1(n = 16)	Group 2(n = 55)	*p*
Gender, male, n (%)	61(85.9%)	15(93.8)	46(83.6)	0.309
Age, years	49.4 ± 12.9	46.6 ± 11.2	50.2 ± 13.4	0.323
BSA (m^2^)	1.85 ± 0.18	1.84 ± 0.17	1.85 ± 0.18	0.917
BMI (kg/m^2^)	26.0 ± 3.3	26.7 ± 2.5	25.8 ± 3.5	0.359
Systolic blood pressure (mmHg)	121 ± 14	118 ± 13	121 ± 15	0.429
Diastolic blood pressure (mmHg)	80 ± 9	78 ± 8	80 ± 9	0.375
Family history of HCM, n (%)	13(18.3)	3(18.8)	10(18.2)	0.959
Family relatives history of HCM, n (%)	18(25.4)	5(31.2)	13(23.6)	0.541
Family history of SCD, n (%)	6(8.5)	1(6.3)	5(9.1)	0.721
NYHA functions				
I, n (%)	69	14	46	
II, n (%)	2	2	0	0.008
≥ III, n (%)	0	0	0	
Symptoms (%)	18(25.4)	14(87.5)	4(7.2)	< 0.001
Chest tightness, n (%)	13	10	3(5.5)	
Chest pain, n (%)	1	1	0	
Palpitation, n (%)	4	3	1(1.8)	
Echocardiography				
Apical HCM, n (%)	3(4.2)	3(18.8)	0	0.001
Suspicious apical HCM, n (%)	8(11.3)	7(43.8) ^*^	1(1.8)	< 0.001
Electrocardiography				
Giant negative T waves, n (%)	71(100)	16(100)	55(100)	1.000
ST-T abnormality, n (%)	15(21.1)	13(81.3)^*^	2(3.6)	< 0.001
LV hypertrophy, n (%)	10(14.1)	9(56.3) ^*^	1(1.8)	< 0.001
Interval (mo) between CMRs	24.4 ± 8.3	33.9 ± 6.4^*^	24.8 ± 5.7	< 0.001
Medications	25	15	10	< 0.001
Beta blocker, n (%)	18	10	8	
Calcium channel blocker, n (%)	3	2	1	
Others, n (%)	4	3	1	

*BSA* body surface area, *BMI* body mass index, *HCM* hypertrophic cardiomyopathy, *SCD* sudden cardiac death, *AHCM* apical hypertrophic cardiomyopathy, *LV* left ventricular; *NYHA* New York Heart Association, *SCD* sudden cardiac death

### Global morphological and functional measures changes

The LA diameter was slightly increased from 32.0 ± 4.4 mm to 32.5 ± 4.4 mm (*p* = 0.002) at follow-up. LVEDVI, LVESVI and LV CI were all decreased (all *p* < 0.05, Table 2). However, LVEF was similar at follow-up CMR examinations (67.4 ± 6.4% vs. 67.8 ± 6.3%, *p* = 0.050). At follow-up, the ApA was decreased 1.8º (*p* = 0.002) when compared with baseline (Table 2). There were no patients with positive LGE at baseline, however, 4 (5.6%) of 71 patients were LGE positive at follow-up CMR (three Group I patients; one Group 2 patient).

**Table 2:** Overall patient global morphological and functional measures derived from CMR

CMR parameters	Baseline (n = 71)	Follow-up (n = 71)	T value	*p*
LA dimension (mm)	32.0 ± 4.4	32.5 ± 4.4	− 3.186	0.002
LVEDD (mm)	47.8 ± 3.7	47.3 ± 4.0	3.216	0.002
LVEF (%)	67.4 ± 6.4	67.8 ± 6.3	− 1.998	0.050
LVEDVI (ml/m^2^)	56.4 ± 11.8	54.5 ± 12.4	4.303	< 0.001
LVESVI (ml/m^2^)	18.6 ± 5.7	17.7 ± 5.5	4.835	< 0.001
LV CI (L/min/m^2^)	2.5 ± 0.6	2.4 ± 1.3	2.557	0.013
BSA(m^2^)	1.85 ± 0.18	1.87 ± 0.18	− 3.690	< 0.001
LV mass(g)	95.6 ± 29.2	99.1 ± 30.6	− 4.657	< 0.001
LV mass index(g/m^2^)	51.7 ± 14.8	52.9 15.3	− 2.846	0.006
ApA (°)	86.3 ± 14.0	84.5 ± 15.6	3.246	0.002
LGE positive(n)	0	4		
LGE mass(g)	0	3.50,3.27,2.63,2.60	− 2.174	0.048
LGE percent (%)	0	4.4,6.2,3.8,4.4	− 2.010	0.033

*LA* left atrium, *LVEDD* left ventricular end diastolic diameter, *LVEF* left ventricular ejection fraction, *LVEDVI* left ventricular stroke volume index, *LVESVI* left ventricle end-systole volume index, *CI* cardiac index, *BSA* body surface area*, LV mass* left ventricular mass*, Max LV wall thickness* maximum left ventricular wall thickness, *LGE* late gadolinium enhancement

When further comparison between the two subgroups was performed, there was no significant difference in all global morphological and functional measures on CMR at baseline (Table 3). The ApA at follow-up in Group 1 was significantly lower than in Group 2. There were significant differences between the two groups in the change of LV mass index and ApA (LV mass index: 1.4 ± 1.9 g/m^2^·yr vs. 0.2 ± 1.2 g/ m^2^·yr, *p* = 0.026; ApA: − 1.5 ± 2.7°/yr vs. − 0.7 ± 2.0°/yr, *p* < 0.001). There was no significant difference in other parameters including LA dimension, LVEDVI and LV CI (all p > 0.05). The detailed global morphological and functional measures changes were presented in Table 3.

**Table 3:** Subgroup analysis of global morphological and functional measures based derived from CMR

	Group 1 (G1)				Group 2 (G2)			*p* for Baseline	*p* for follow-up	*p* for change
	Baseline (n = 16)	Follow-up (n = 16)	Change /years		Baseline (n = 55)	Follow-up (n = 55)	Change /yr			
LA dimension (mm)	32.4 ± 5.2	33.5 ± 4.6	0.4 ± 0.8		31.9 ± 4.2	32.2 ± 4.4	0.2 ± 0.5	0.679	0.319	0.211
LVEDD (mm)	47.8 ± 3.8	46.6 ± 5.1	− 0.5 ± 0.8		47.8 ± 3.7	47.5 ± 3.7	− 0.2 ± 0.6	0.991	0.474	0.101
LVEF (%)	67.5 ± 6.4	69.5 ± 5. 9	0.7 ± 0.9		67.4 ± 6.4	67.4 ± 6.4	0.0 ± 0.8	0.938	0.243	0.002
LVEDVI (ml/m^2^)	56.3 ± 14.3	52.9 ± 17.1	− 1.3 ± 2.4		56.4 ± 11.1	55.0 ± 10.9	− 0.9 ± 1.3	0.968	0.569	0.542
LVESVI (ml/m^2^)	18.3 ± 5.8	16.1 ± 5.0	− 0.8 ± 0.9		18.6 ± 5.8	18.1 ± 5.6	− 0.3 ± 0.4	0.851	0.188	0.039
LV CI (L/min/m^2^)	2.50 ± 0.66	2.50 ± 0.83	− 0.01 ± 0.14		2.47 ± 0.55	2.39 ± 0.55	− 0.04 ± 0.09	0.726	0.550	0.334
LV mass(g)	97.1 ± 26.1	106.5 ± 29.5	3.3 ± 3.1		95.2 ± 30.2	96.9 ± 30.9	1.2 ± 2.4	0.822	0.276	0.005
LV mass index(g/m^2^)	52.8 ± 13.8	56.8 ± 14.6	1.4 ± 1.9		51.4 ± 15.2	51.7 ± 15.5	0.2 ± 1.2	0.750	0.245	0.026
apA (°)	76.3 ± 17.2	72.7 ± 21.5	− 1.5 ± 2.7		89.2 ± 11.5	87.9 ± 11.6	− 0.7 ± 2.0	0.001	< 0.001	< 0.001
LGE positive(n)	0	3	–		0	1	–	–	–	–
LGE volume(g)	0	3.50,3.27,2.60	–		0	2.63	–	–	–	–
LGE percent (%)	0	4.4,6.2, 4.4	–		0	3.8,	–	–	–	–

P < 0.05 is considered significant. *LAD* left atrium dimension, *LV*  left ventricular, *LVEDD* left ventricular end diastolic diameter, *LVEF* left ventricular ejection fraction, LVEDVI left ventricular stroke volume index, *LVESVI* left ventricle end-systole volume index, *CI* cardiac index, *LGE* late gadolinium enhancement

### Follow-ups of the distribution of segmental wall thickness

We studiedchanges of LV wall thickness in the basal (segment 1–6), middle (segment 7–12) and apical (segment 13–16) segments based on the AHA 17-segment model. Compared with the baseline exam, the basal, mid, and apical LV wall thickness were all increased by a mean of 0.3 mm, 0.2 mm and 0.8 mm, respectively (all *p* < 0.001). Further subgroup analysis demonstrated that there were no significant differences in LV wall thickness at basal and middle parts at baseline or at follow-up (all p = NS). However, the mean apical wall thickness was significantly different between the two groups at both baseline and at follow-up, with Group 1 have greater wall thickness at both time points (all *p* < 0.001, Table 4). There were significant difference in wall thickness changes between the two groups at basal, mid, and apical (all *p* < 0.05), and the changes in apical wall thickness was the most significant(0.75 ± 0.16 mm/yr vs. 0.26 ± 0.14 mm/yr, *p* < 0.001).

**Table 4:** Subgroup analysis of the changes of wall thickness in basal, mid and apical left ventricle

	Group 1(G1)			Group 2(G2)			*p* for Baseline	*p* for Follow-up	*p* for change
	Baseline (n = 16)	Follow-up (n = 16)	Change /yr	Baseline (n = 55)	Follow-up (n = 55)	Change /yr			
Basal (mm)	8.3 ± 0.9	8.5 ± 0.9	0.09 ± 0.03	7.9 ± 0.9	8.1 ± 0.9	0.12 ± 0.05	0.167	0.127	0.003
Middle (mm)	7.5 ± 1.2	7.7 ± 1.2	0.08 ± 0.02	7.0 ± 1.2	7.2 ± 1.3	0.11 ± 0.06	0.122	0.109	0.002
Apical (mm)	8.6 ± 1.0	10.7 ± 1.2	0.75 ± 0.16	7.0 ± 1.1	7.5 ± 1.2	0.26 ± 0.14	< 0.001	< 0.001	< 0.001

P < 0.05 is considered significant. Basal = mean thickness of segment 1–6, Middle = mean thickness of segment 7–12, Apical = mean thickness of segment 13–16

**Fig. 3:**
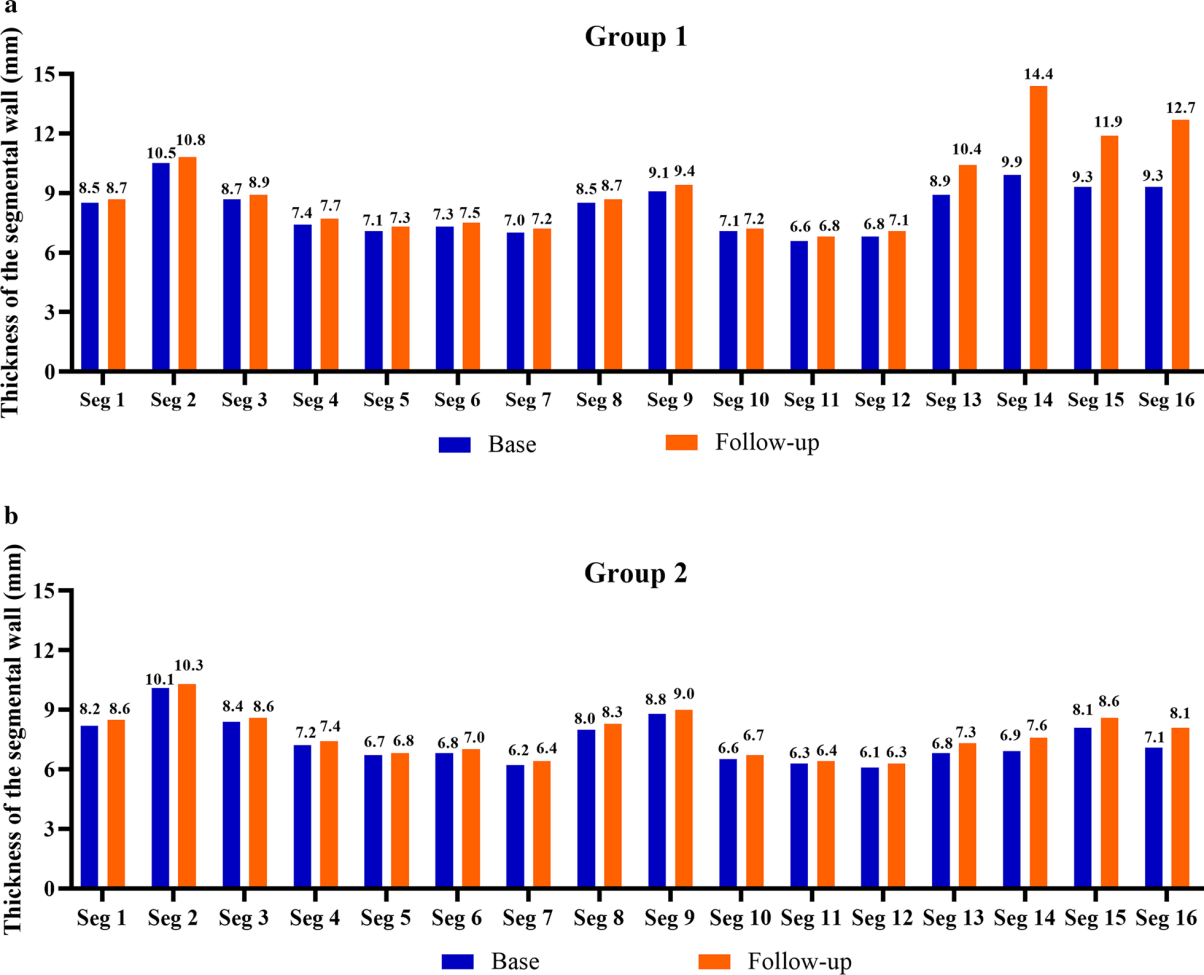
The head-to-head comparison of the changes of all 16-segmental thicknesses of the left ventricle (LV) in patients who did not meet HCM criteria (wall thickness ≥ 15 mm without family history or ≥ 13 with a family history of HCM) at baseline or at follow-up between group 1 (**a**) and group 2 (**b**). at follow-up, the thickness of all LV segments significantly increased. The thickness of apical portion (segment 13–16) increased the most. *Seg* segment

**Fig. 4:**
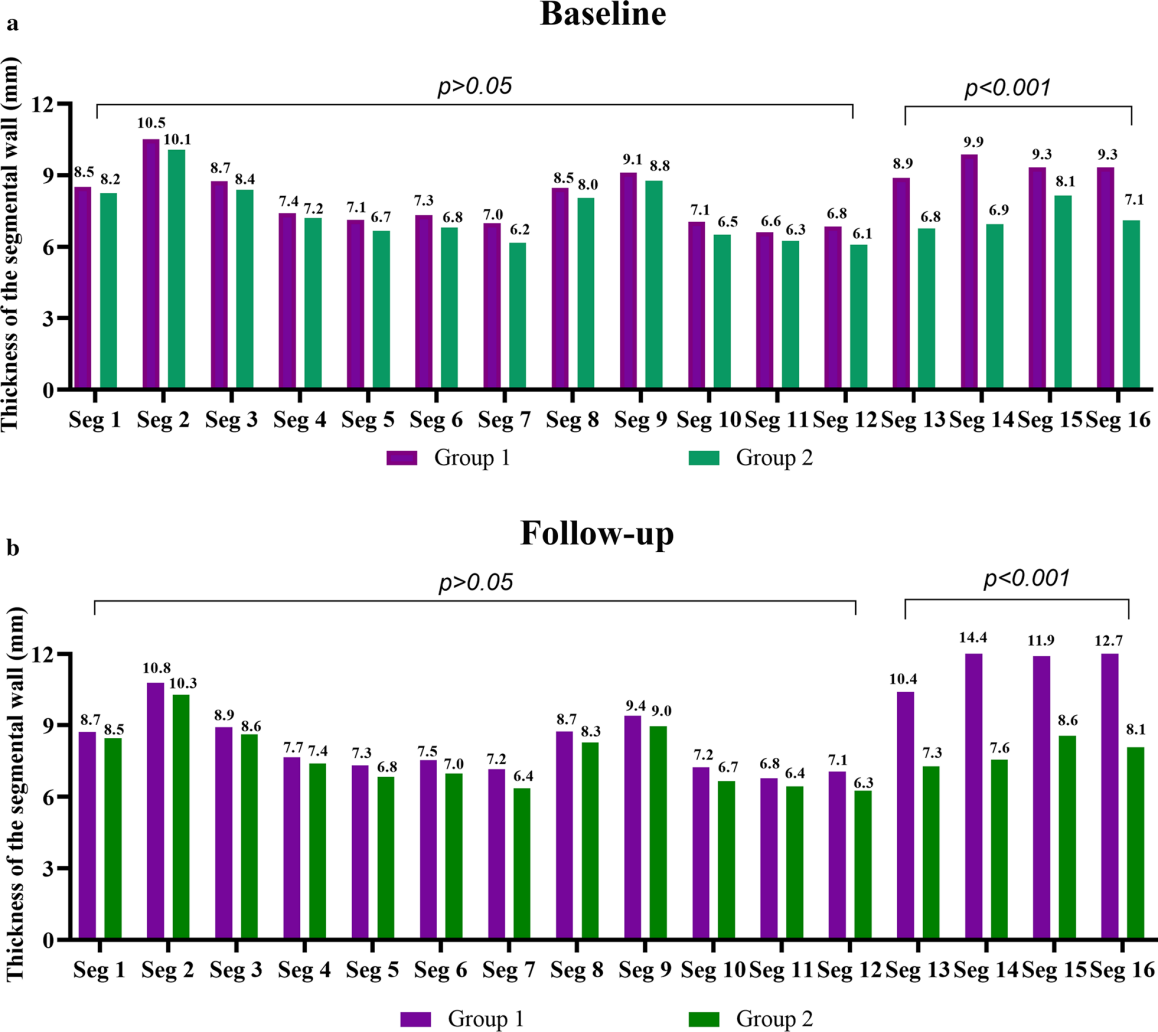
Comparison of LV wall thickness for all 16 segments in patients who had apical wall thickness < 15 mm without a family history or < 13 with a family history of HCM) at baseline (**a**) and at follow-up (**b**) for both groups. There were no significant differences both in basal (seg 1–6) and middle (seg 7–12) at either baseline and follow-up. However, there were significant differences in apical segments (seg 13–16) both at baseline and follow-up. *FH* family history, *Seg* segment

Comparison of the segmental thickness between baseline and follow-up using the AHA 17-segmental LV model, similar results were obtained. Regardless of the overall or subgroup analysis, at follow-up, LV segment wall thickness increased when compared to baseline (Fig. 3). The thickness of apical portion (segment 13–16) increased greatest. However, further comparing the segmental wall thickness at baseline among the subgroups, only the apical segments were significant (segment 13–16, p < 0.001, Fig. 4).

### CMR predictors of apical HCM in patients with giant T wave inversion

**Fig. 5:**
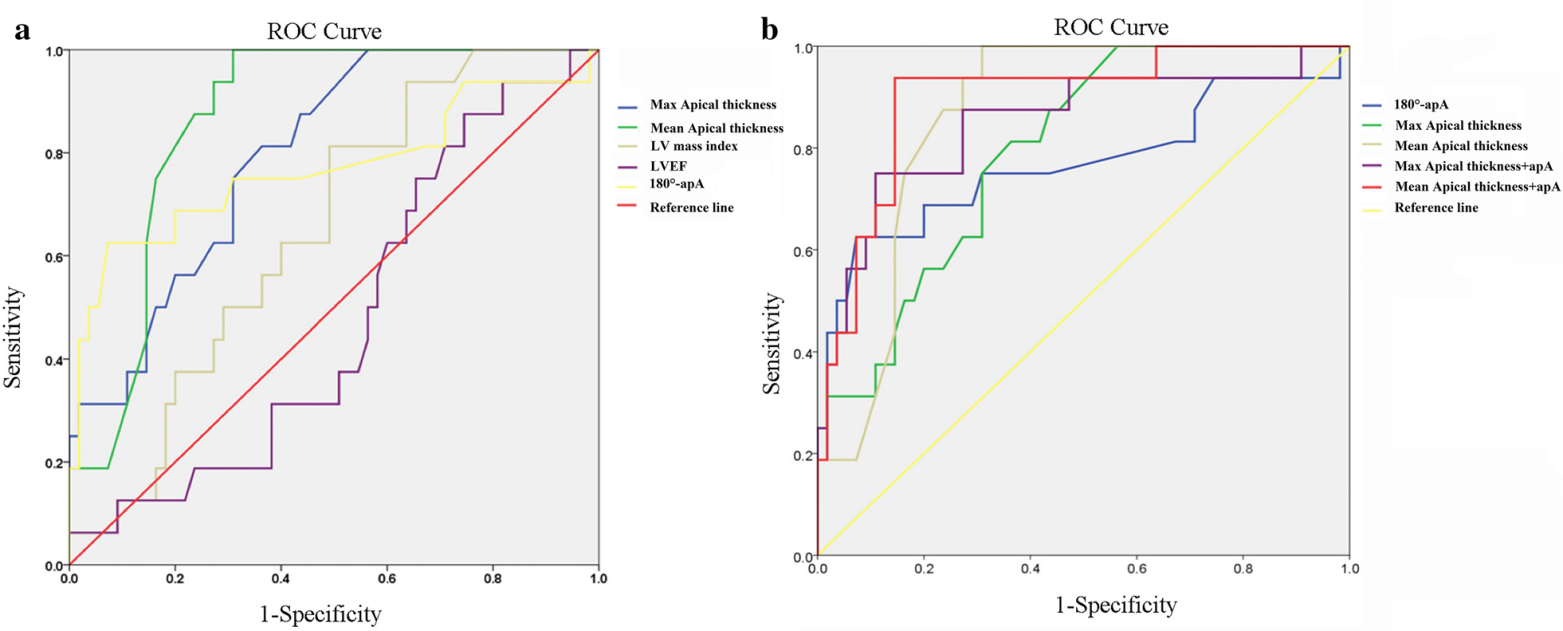
Receiver operating curves (ROC) showed that the predictive performance of single (**a**) and joint (**b**) CMR parameters for apical HCM. Mean apical thickness (+ ApA) is the best predictor for apical HCM, which had an AUC of 0.898, a sensitivity of 93.8%, and a specificity of 85.5% (p < 0.001). *Max* maximum, *LV* left ventricular, *LVEF* left ventricular ejection fraction

**Fig. 6:**
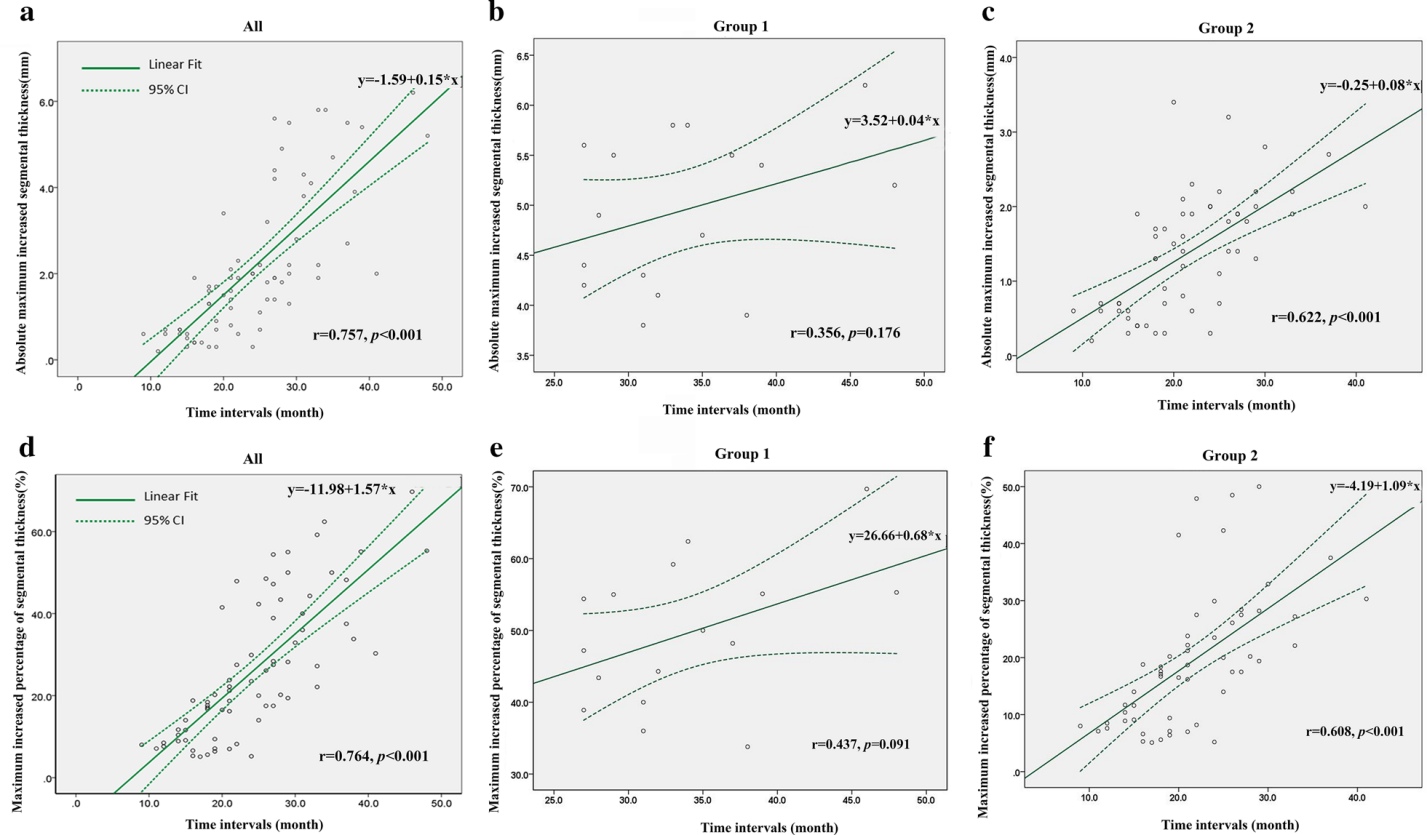
Relationship between the time intervals of CMR examinations and the maximum absolute increase in LV wall thickness in all patients (**a**), Group 1 (**b**) and Group 2 (**c**). The relationship between time intervals of CMR examinations and maximum percentage of increased segmental thickness at the apex (segmental 13–16) is shown in all patients (**d**), Group 1 (**e**) and Group 2 (**f**). CI indicates confidence interval

For apical HCM prediction analysis, area under curve (AUC) of the ROC were 0.87 (95% confidence interval, CI, 0.78–0.95, *p* < 0.001) in mean apical thickness at baseline, 0.80 (95% CI, 0.69–0.91, *p* < 0.001) in maximum apical segmental thickness, and 0.77 (95% CI, 0.61–0.93) in ApA, respectively. Further analysis indicated that the cutoff thickness of 7.6 mm (mean apical thickness at baseline) yielded a sensitivity of 100% and a specificity of 69%; the cutoff thickness of 9.5 mm (maximum apical segmental thickness) yielded a sensitivity of 81% and specificity of 64%; and a cutoff of 75° of ApA yielded a sensitivity of 63% and specificity of 93% for prediction of development to fulfill criteria for apical HCM (Fig. 5). Our results showed that mean apical thickness + ApA was the best predictor for progression to apical HCM (AUC = 0.898), which was a little higher than mean apical thickness at baseline (AUC = 0.865). Utilizing both the LV wall thickness and the ApA together best identifies/predicts apical HCM. Both the absolute maximum wall thickening and the maximum percentage of wall thickening were related to the time interval between CMR examinations for the whole cohort (Fig. 6). Further univariable Cox regression model indicated ApA and the change rate of apical thickness were associated with the development of apical HCM (Table 5).

**Table 5:** Univariable analysis of CMR characteristics for prediction of apical hypertrophic cardiomyopathy in patients with unexplained giant T-wave inversion

Baseline variables	Typical apical HCM (n = 16)	Non-apical HCM (n = 55)	HR (95% CI)	p value
Age (years)	15 (93.8)	46 (83.6)	1.014 (0.963, 1.068)	0.595
Male sex, n (%)	46.6 ± 11.2	50.2 ± 13.4	8.427 (0.874, 81.232)	0.065
Apical thickness (mm)	8.6 ± 1.0	7.0 ± 1.1	1.425 (0.915, 2.220)	0.118
Change rate of apical thickness(mm/y)	0.75 ± 0.16	0.26 ± 0.14	1.084 (1.032, 1.139)	0.001
Apical angle (°)	76.3 ± 17.2	89.2 ± 11.5	0.954 (0.918, 0.991)	0.016
Change rate of apical angle(°/y)	− 1.5 ± 2.7	− 0.7 ± 2.0	0.779(0.589, 1.031)	0.081

*HCM* hypertrophic cardiomyopathy, *HR* hazard ratio

### Reproducibility tests

**Fig. 7:**
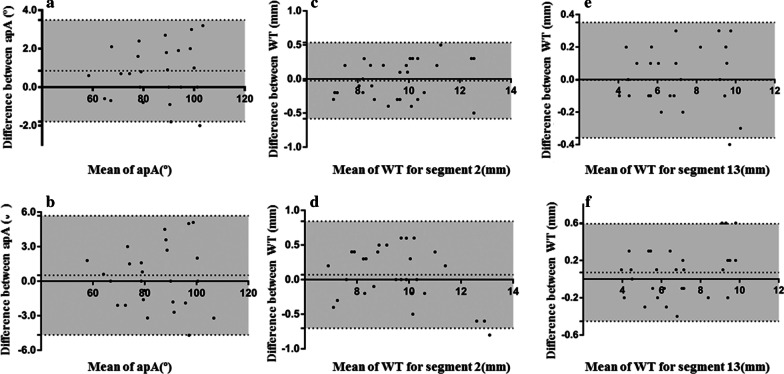
Bland Altman analysis of the apical angle (ApA), wall thickness (WT) of segment 2, and wall thickness for segment 13 for intra- and inter-observer variability in this cohort. Variability of intra-observer (**a**) and inter-observer (**b**) for ApA, intra-observer (**c**) and inter-observer (**d**) for segment 2 wall thickness, and intra-observer (**e**) and inter-observer (**f**) for segment 13 are shown. The middle-dashed line indicates the mean difference and gray area between dash lines indicates the standard deviation

Interobserver and intraobserver variability were tested in a sub-group of 30 randomly selected patients. Measurements of ApA, LV wall thickness of segment 2, and LV wall thickness of segment 13 were displayed on a Bland–Altman plot (Fig. 7). ApA, wall thickness of segment 2, and wall thickness of segment 13 had an intraobserver variability of 0.7 ± 1.3º, − 0.0 ± 0.3 mm and − 0.0 ± 0.2 mm, and an inter-observer variability of 0.5 ± 2.6º, 0.1 ± 0.4 mm and 0.1 ± 0.3 mm, respectively. Inter-observer agreements were very high for ApA (ICC = 0.977, 95% CI 0.952–0.989), wall thickness of segment 2 (ICC = 0.971, 95% CI 0.941–0.986) and segment 13 (ICC = 0.990, 95% CI 0.979–0.995). Intra- observer were also high for ApA (ICC = 0.994, 95% CI 0.987–0.997), wall thickness of segment 2(ICC = 0.983, 95% CI 0.965–0.992) and wall thickness of segment 13 (ICC = 0.996, 95% CI 0.991–0.998).

## Discussion

This study is a follow-up to our previous CMR study in which we had identified a special group of patients who did not meet criteria for typical apical HCM, but had a collection of characteristics suggestive of disease akin to apical HCM which included abnormal apical morphology (lack of thinning of the apical myocardium relative to the basal myocardium) and unexplained giant T-wave inversion on ECG. To the best of our knowledge, this is the first study to follow-up the abnormal CMR morphological features of these patients. Our results demonstrated several important findings. First, during a mean 2 year follow-up, over one-fifth of these patients progress to fulfill criteria for apical HCM. This finding provides convincing CMR-based evidence that this patient group could be considered to have an early or pre-apical HCM phenotype [9]. Second, although not all the subjects progressed to fulfill apical HCM criteria, LV segmental thickness did progress over time, particularly within the apical segments. This suggests that this special group of patients was different from normal subjects. Third, according to our subgroup analysis, patients with an average apical wall thickness that is thicker at baseline are more likely to progress to typical apical HCM in a relatively shorter period of time. The combination of the mean apical thickness at baseline and ApA is the best predictor for apical HCM (threshold of 90°and 8.1 mm, respectively) and had a sensitivity of 93.8% and a specificity of 85.5%. Because only a few subjects' baseline echocardiography indicated apical HCM (3 confirmed diagnosis and 8 suspected diagnoses), our findings provide important information that can make an early diagnosis of apical HCM.

### Variability in left ventricular global morphological and functional results

As of now, there have been no studies of the segmental LV thickness of normal, healthy subjectsand only a few studies on the overall LV mass in normal subjects [18–21]. A longitudinal CMR LV mass study was reported by Moody et.al. in 42 healthy subjects with CMR at baseline and a 1-year [20]. They found no significant changes in any LV parameter (LV mass decreased by 2.0 ± 8.6 g; p = 0.014). Another study by Maceira et al. studied 120 healthy subjects grouped by age and gender and found that LV mass tends to decrease with age in males, but increased in females [19]. In our current study, we found that both LV mass and LV mass index increased after 24 months, which differs from studies of normal subjects. The ApA was a novel parameter introduced in our previous study [9]. In this study, we found that ApA is a useful parameter not only to detect apical HCM, but also predict development of apical HCM phenotype. We found that the ApA was significantly decreased not only in the cohort but also in a further subgroup analysis (Groups 1 and 2). The decrease rate of APA in Group 1 was faster than that in Group 2. A threshold value of 75° of the ApA yielded a sensitivity of 62.5% and specificity of 92.7% for the prediction of apical HCM. Our previous study showed a significantly decreased ApA in patients who had a deep T-wave inversion, but did not meet any criteria for apical HCM. The ApA is a sensitive variable as it can change even if the LV hypertrophy is confined to a small region. Therefore, all our findings regarding ApA add important evidence-based medical data for its clinical application in predicting and diagnosing apical HCM.

### Variability in segmental wall thickness

Traditionally, apical HCM was diagnosed in patents with an apical wall thickness ≥ 15 mm (or ≥ 13 with family history of HCM) at end-diastole based on the guidelines recommended by AHA/ACCF[7], or ESC[8]. Until now, there were no other diagnostic criteria for apical HCM. Both our previous study as well as another additional study suggested that an apical wall thickness of 12 to 15 mm, or a ratio of apical maximal thickness to basal inferolateral wall ≥ 1.3 may represent early findings of apical HCM[9, 10]. Our previous study has also showed that the apical morphology in patients who did not meet criteria for apical HCM but had unexplained giant T-wave inversion differed from normal subjects. Specifically, the LV apex in these patients lacked the normal thinning of myocardium relative the basal segments of myocardium when compared with healthy subjects. In our current follow-up study, we found progression of the apical abnormalities in this patient group. Although the follow-up time of group 1 was longer than that of group 2, the result showed that the change rate of apical thickness was still significant between the two groups. Combining the findings of our current study with findings in our prior study, we conclude that a large minority of patients who do not meet criteria for apical HCM, but lack the progressive thinning of myocardium from the base to the apex and have giant T-wave inversions on ECG, will have phenotypic progression over time to meet criteria for apical HCM.  Though unproven, it is likely that more patients would meet criteria with further observation. 

### Potential, mild, or pre-AHCM

It has been reported that even early, mild apical hypertrophy (lack of apical thinning) without the classic spade configuration presents with giant negative T waves on ECG [5, 22]. From the results of our limited time of follow-up duration, we believe this represents a pre-clinical apical HCM. Perhaps a new variant “pre-apical HCM” may be an appropriate diagnosis for this subset of patients presenting with giant T-wave inversions and mild abnormal apical morphology and time may be an important factor in these patients. Univariate analysis shows it is the change rate of apical thickness rather than apical thickness at baseline that is closely related to the development of apical HCM. In the current study, the absolute increase in thickness of the apical wall as well as the percentage of the wall thickness increase strongly correlated with the duration of interval time between the two CMR examinations. Therefore, we may speculate that as the follow-up time increases, more patients will declare themselves as typical, apical HCM. However, the LV wall thickness of normal human beings also increase with age [23]. In contrast, the ApA at baseline seems to be more convenient than the annual change of apical wall thickening, we may predict the progression of apical HCM from the ApA at baseline. Due to the limitation of sample size, multivariate analysis was not appropriate in this study. Therefore, larger sample size and longer follow-up investigation is needed to evaluate the outcome of these patients.

### Limitations

Our study has several limitations. First, genetic testing was not routinely obtained, but does not affect our primary conclusion. However, HCM-related gene variants and their clinical outcome have been shown to be inconsistent due to heterogeneity of both genotype and phenotype [1, 24, 25]. For this reason, genetic testing is only recommended for the screening of relatives of positive HCM cases [1, 8]. The diagnosis of HCM remains largely clinical and largely relies on non-invasive testing. Second, the relatively small number of patients enrolled in a single center, together with the relatively short duration of follow-up time represents an obvious limitation. It is possible that pre-apical HCM patients in Group 2 would have been classified as Group 1 if the CMR interval had been longer. Prospective studies in large patient population are needed to further validate our results, especially to verify the threshold values for the segmental thickness of LV wall and ApA in patients who have giant T-wave inversion but do not meet criteria for typical apical HCM. Further work will be able to translate how these measurements could be implemented into daily clinical practice.

## Conclusions

Morphological and functional changes occur in patients who did not meet apical HCM criteria but have baseline abnormal LV apical morphology and unexplained giant T-wave inversions on ECG. More than one-fifth of these patients progress to typical apical HCM on CMR and another half will have a significant (> 15%) increase of LV apical wall thickness. The combination of mean apical wall thickness and ApA is the best predictor for development of apical HCM with a threshold value of 90° and 8.1 mm yielding a sensitivity of 93.8% and a specificity of 85.5%. Further prospective studies and longer follow-up are needed to further validate our results.

## Supplementary information

**Additional file 1.** morphological characteristics of normals on CMR. As shown in this figure, considerable variation in LV wall thickness was observed with progressive thinning from the base to apex. (A–C) Location of basal and middle LV slices for measurement. Apical segments were measured on two- and four-chamber views (D and E). Three measurements were taken at the thickest region of each segment (C and D), and then the average was collected. Two 1-cm-long lines were drawn out from the apex vertex to bilateral endocardium, and the angle formed was apA (E).

## Data Availability

The datasets used and/or analyzed during the current study available from the corresponding author on reasonable request.

## Abbreviations

ACCF: American College of Cardiology Foundation
AHA: American Heart Association
ApA: Apical angle
AUC: Area under the curve
BMI: Body mass index
BSA: Body surface area
bSSFP: Balanced steady state free precession
CI: Cardiac index
CMR: Cardiovascular magnetic resonance
ECG: Electrocardiogram
ESC: European Society of Cardiology
FH: Family history
FOV: Field of view
HCM: Hypertrophic cardiomyopathy
iPAT: Integrated parallel imaging technique
LA: Left atrium/left atrial
LGE: Late gadolinium enhancement
LV: Left ventricle/left ventricular
LVEDD: Left ventricular end-diastolic diameter
LVEDV: Left ventricular end-diastolic volume
LVEDVI: Left ventricular end-diastolic volume index
LVEF: Left ventricular ejection fraction
LVESV: Left ventricular end-systolic volume.
LVESVI: Left ventricular end-systolic volume index
PSIR: Phase sensitive inversion recovery
WT: Wall thickness
