# Supplementary material for: Patients who do not fulfill criteria for hypertrophic cardiomyopathy but have unexplained giant T-wave inversion: a cardiovascular magnetic resonance mid-term follow-up study
Source: J Cardiovasc Magn Reson. 2021 Jun 3;23:67. doi: 10.1186/s12968-020-00700-5 (PMC8173876; doi:10.1186/s12968-020-00700-5)
Supplement: Supplementary file 1 — Additional file 1. morphological characteristics of normals on CMR. As shown in this figure, considerable variation in LV wall thickness was observed with progressive thinning from the base to apex. (A–C) Location of basal and middle LV slices for measurement. Apical segments were measured on two- and four-chamber views (D and E). Three measurements were taken at the thickest region of each segment (C and D), and then the average was collected. Two 1-cm-long lines were drawn out from the apex vertex to bilateral endocardium, and the angle formed was apA (E). [file 12968_2020_700_MOESM1_ESM.docx]

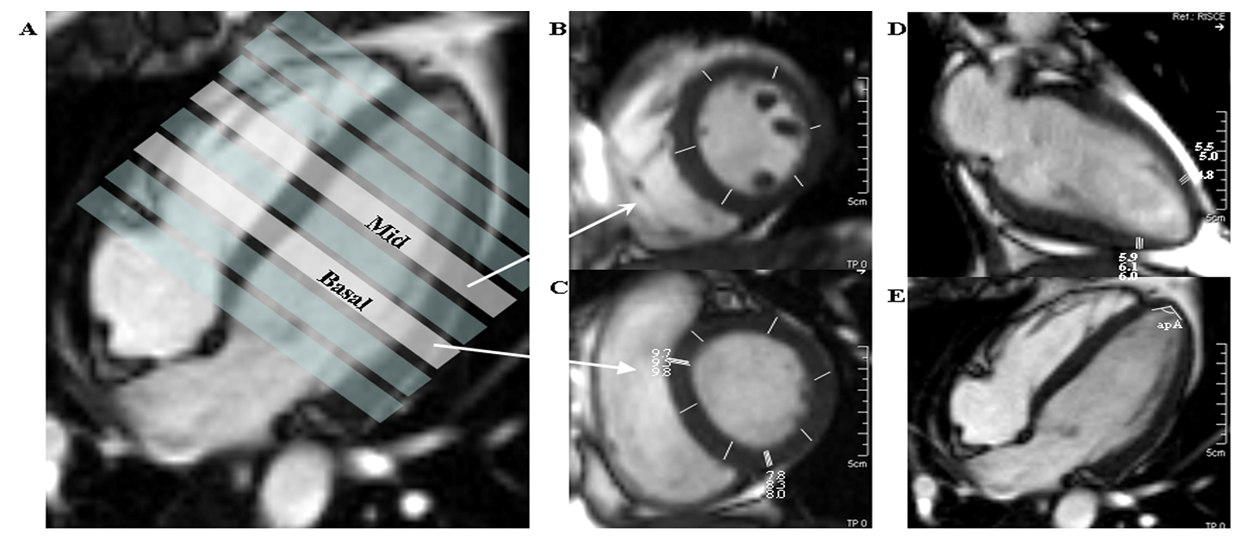


Figure S1: Measurements of LV segmental wall thickness and apical angle(apA) in a normal subject. Considerable variation in LV wall thickness was observed with progressive thinning from the base to apex. (A–C) Location of basal and middle LV slices for measurement. Apical segments were measured on two- and four-chamber views (D and E). Three measurements were taken at the thickest region of each segment (C and D), and then the average was collected. Two 1-cm-long lines were drawn out from the apex vertex to bilateral endocardium, and the angle formed was apA (E).^1^

1. Wu B, Lu M, Zhang Y, Song B, Ling J, Huang J, Yin G, Lan T, Dai L, Song L, Jiang Y, Wang H, He Z, Lee J, Yong HS, Patel MB and Zhao S. CMR assessment of the left ventricle apical morphology in subjects with unexplainable giant T-wave inversion and without apical wall thickness >/=15 mm. *European heart journal cardiovascular Imaging*. 2017;18:186-194.
